# Severity of irritable bowel syndrome and its correlates among Middle East and North African medical students: a multicentric cross sectional study

**DOI:** 10.1038/s41598-026-69035-y

**Published:** 2026-09-09

**Authors:** Abdelrahman Abdelshafi, Zeinab G. Abdelhamid, Belal Osama, Yasmine Adel Mohammed, Nehal Allam, Youssef Gouda Youssef, Hamza A. Abdul-Hafez, Sara Sabbagh, Ibrahim Abdelnasar Yakout, Ziad Aljarad, Doaa Mohamed Osman, Doaa Mazen Abdel-Salam

**Affiliations:** 1 Faculty of Medicine Assiut University, Assiut, Egypt; 2 Department of Public Health and Community Medicine, Faculty of Medicine, Assiut University, Assiut, Egypt; 3 Faculty of Medicine, Benha University, Benha, Egypt; 4 Department of Pharmacology, Faculty of Medicine, Assiut University, Assiut, Egypt; 5 Oncology Center of Mansoura University, Mansoura, Egypt; 6 Department of Medicine, Faculty of Medicine and Health Sciences, An-Najah National University, Nablus, Palestine; 7 Faculty of medicine, Damascus University, Damascus, Syria; 8 Faculty of Pharmacy, Zagazig University, Shaqia, Egypt; 9 University of Aleppo, Aleppo, Syrian Arab Republic

**Keywords:** Irritable bowel syndrome (IBS), MENA region, Correlates, Anxiety, Depression, IBS-SSS (IBS Symptom Severity Scale), Diseases, Gastroenterology, Health care, Medical research, Psychology, Psychology

## Abstract

Irritable bowel syndrome (IBS) is a prevalent functional gastrointestinal disorder that is commonly reported among individuals experiencing high levels of stress, such as medical students. Despite its significance, data on the severity of IBS and its correlates remains scarce in the Middle East and North Africa (MENA) region. This study is a secondary analysis of a previously published multicenter study and aimed to evaluate the severity of irritable bowel syndrome (IBS) and identify the correlates of severe IBS among medical students with Rome IV-confirmed IBS. This multicentric cross-sectional study was conducted across 27 medical faculties in seven MENA countries) from March until May 2024. A bilingual (Arabic/English) online self-reported questionnaire was used to assess IBS severity via the validated IBS-Symptom Severity Scale. Data analysis was performed using SPSS, version 26. Remission, mild, moderate, and severe IBS symptoms were detected among 15.28%, 31.38%, 36.60%, and 16.73% of the participants, respectively. Students with severe IBS symptoms had a significantly higher mean age compared to other students (*p* = 0.019). Among students with severe IBS symptoms, the proportion of overweight/obese (18.5%) was significantly higher than that of underweight/normal weight (16.1%) (*p* = 0.022). Furthermore, among students with severe IBS symptoms, there were significantly higher proportions of positive depression (*p* < 0.001), anxiety (*p* < 0.001), and history of food or drug hypersensitivity (*p* < 0.001). In the Generalized linear model – Adjusted Poisson regression analysis for determination of the correlates of severe IBS among medical students, Anxious students [aPR = 1.87 (95% CI 1.418–2.461)] and depressed students [aPR = 1.86 (95% CI 1.512–2.284)] were significantly more likely to experience severe IBS symptoms (*p* < 0.001*). Similarly, students with a history of food or drug hypersensitivity were significantly more likely to have severe IBS [aPR = 1.29 (95% CI 1.079–1.546)] (*p* = 0.005). The present study demonstrated that more than half of students with IBS In the MENA region exhibited moderate to severe symptoms. Psychological comorbidities were predominant among students with severe IBS symptoms, highlighting the strong association between mental health conditions and IBS symptom severity in this population.

**Supplementary Information:**

The online version contains supplementary material available at https://doi.org/10.1038/s41598-026-69035-y.

## Background

Irritable bowel syndrome (IBS) is a prevalent functional gastrointestinal disorder distinguished by altered bowel movements, changes in stool form, abdominal distention, as well as abdominal discomfort or pain. This chronic disease has no identifiable organic etiology, structural underlying abnormalities, or evident diagnostic biomarkers; therefore, the diagnosis mainly depends on clinical assessment^1,2^. On this basis, the Rome IV IBS diagnostic criteria showed adequate sensitivity and excellent specificity, providing that symptoms should have appeared no less than six months before setting up the diagnosis of IBS, including recurrent abdominal pain at least one day a week in the last three months, accompanied by two or more other IBS symptoms^3^.

However, many biological, psychological, and social factors were associated with pathogenesis of this illness, including gender, dietary regimen, stressful lifestyles and several psychiatric disorders, making IBS adherent to a biopsychosocial model^4,5^. Within this framework, the stress-diathesis model suggests that psychological distress acts as a primary catalyst for symptom exacerbation in vulnerable individuals. This exacerbation is mechanistically driven by the dysregulation of the hypothalamic-pituitary-adrenal (HPA) axis and subsequent neuroendocrine alterations, which collectively induce visceral hypersensitivity and altered gastrointestinal motility^6^. Furthermore, contemporary literature on the microbiome-gut-brain axis highlights how chronic academic and psychological stress can alter gut microbiota composition, creating a neuroinflammatory feedback loop that bridges central nervous system distress with peripheral gastrointestinal symptom severity^7,8^.

Psychiatric comorbidity, in particular, is prevalent among patients with IBS, as psychosocial factors and its association with gastrointestinal symptoms, illness experience, and clinical outcomes, which is strongly associated with reduced quality of life^9^. Consequently, stressful experiences have been associated with the presence and greater severity of functional gastrointestinal disorders^9^. Previous studies have shown a wide range of IBS prevalence that varies significantly depending on the geographical region, study methodology, and the specific diagnostic criteria applied. Most notably, the landmark Rome Foundation Global Study conducted by Sperber et al. across 33 countries demonstrated that the worldwide prevalence of IBS was 10.1% when using the Rome III criteria, but adjusted to a significantly lower 4.1% when applying the more stringent Rome IV criteria^10^. Furthermore, while historical studies on the general population of Western countries often estimated prevalence rates between 10% and 20%, contemporary global data underscores that tightening the diagnostic threshold for abdominal pain frequency under Rome IV yields more conservative, yet clinically severe, estimates^10^. Since university students are highly exposed to stressful conditions such as high workload, academic stress, exam-related anxiety, financial struggles, interpersonal conflicts, career choices, life planning and life goal decisions, some studies aimed to assess IBS symptoms prevalence among this population^4^.

Medical school is widely recognized as highly stressful and demanding; consequently, medical students experience greater stress levels than the general population and are therefore at an increased risk of developing IBS compared with students in other disciplines^11,12^. All these considerations have made it of great importance to study IBS among this population. Studies conducted among medical students at the University of Sharjah, UAE^1^, and Bangladesh^11^, reported IBS prevalence rates of 17.3% and 22.88%, respectively.

In the same context, a meta-analysis study on anxiety and depressive symptoms in IBS patients has demonstrated prevalence rates of 39.1% and 28.8% respectively^13^.

All the points mentioned above demonstrate that the severity of IBS is strongly associated with the well-being of medical students. Accordingly, the IBS severity scoring system (IBS-SSS) was designed to assess the severity and frequency of IBS symptoms, including abdominal pain and bloating, as well as the patients’ satisfaction with their bowel habits and the interference of abdominal discomfort and altered bowel functioning with their daily lives^14^.

Beyond overall prevalence, understanding the spectrum of symptom severity is equally critical; comprehensive systematic reviews, such as those by Farrukh and colleagues, have established that psychological distress and psychiatric comorbidities are primary predictors of more severe IBS trajectories^15^.

Because of the lack of comparable studies, limited previous samples, and the gravity of this topic, this multicentric cross-sectional study was conducted to evaluate the severity of IBS symptoms among medical students in the MENA (Middle East and North Africa) region, trying to shed light on this common disorder among such a highly stressed population, and ultimately contributing to improve their overall well-being and academic performance. To the best of our knowledge, this is the first large multicentric study to characterize IBS symptom severity and its associated factors among medical students across multiple countries in the MENA region. Although the observed associations are consistent with previous international evidence, this study provides region-specific estimates that may inform student health policies and medical education initiatives within the MENA context.

## Subjects and methods

### Study site, design and population

This study represents a secondary analysis of a previously published multicenter cross-sectional dataset of medical students from seven Middle East and North African (MENA) countries^16^. It aimed to evaluate IBS symptom severity using the validated IBS Symptom Severity Scale (IBS-SSS) and to identify the correlates of severe IBS among students with Rome IV-confirmed IBS, thereby extending the findings of the original prevalence study.

The parent study recruited 8275 medical students from 27 faculties of medicine across seven countries (Egypt, Palestine, Syria, Jordan, Iraq, Libya, and Sudan) between March and May 2024. IBS was diagnosed according to the ROME IV criteria using a validated self-administered questionnaire^3^.

For the present study, the authors restricted the analysis in the original data set to medical students classified as IBS-positive (*n* = 3317). Sociodemographic characteristics and IBS prevalence data were extracted from the parent study, while additional variables were analyzed specifically within the IBS-positive subgroup. More detailed information about parent study has been described elsewhere^16^.

### Inclusion and exclusion criteria

Participants included in this analysis were those identified as IBS-positive in the original dataset. The exclusion criteria applied in the parent study include prior diagnoses of inflammatory bowel disease, gastroesophageal reflux disease, gastrointestinal red-flag symptoms, and a family history of gastrointestinal cancers.

### Sample size calculation

Sample size of the parent study was originally calculated using the Epi info version 7 StatCalc, based on the following assumption: prevalence rate of severe IBS among IBS positive patients (14.3%)^17^, level of confidence 99%, precision 5%, and design effect 1. The minimum estimated sample size is 325. After adding 10% as non-response rate, the sample size was raised to 358. The authors included all IBS positive medical students from the original data (*n* = 3317) in the analysis. The number of medical students included in this study exceeded the minimum required estimated sample size.

### Sampling technique and data collection approach

In the parent study, a multistage approach was applied to ensure geographic and institutional diversity. First, simple random sampling was used for selecting 27 universities from a list of all medical faculties in the seven MENA countries. Second, within each selected university, a convenience sampling approach was used for inviting medical students to participate primarily via social media platforms. A third stage was conducted in the current study, involving complete coverage of all medical students who tested positive for IBS.

### Data collection tool

The authors distributed a self-reported online questionnaire, which was available in both Arabic and English languages. Each item of the survey assessed a specific aspect associated with the study aims. The distributed questionnaire composed of different parts as follows; sociodemographic characteristics, nutritional habits, personal behaviors, The Rome IV diagnostic questionnaire for adults with IBS, Generalized Anxiety Disorder seven-item (GAD-7) questionnaire, and Patient Health Questionnaire (PHQ-9). Detailed description of the scales and their scoring system were reported elsewhere^16^.

In addition, the questionnaire included Irritable Bowel Syndrome Symptom Severity Scale (IBS-SSS). It is a widely used tool to assess the severity of IBS symptoms^16,17^.

The IBS-SSS questionnaire measures five key aspects: the intensity of abdominal pain, frequency of abdominal pain, the degree of abdominal distension, dissatisfaction with bowel habits, and the overall impact of IBS on daily life. Each item is scored on a visual analog scale ranging from 0 to 100. The total IBS-SSS score was categorized into remission (< 75), mild (75–<175), moderate (175–<300), and severe (≥ 300)^14^. The IBS-SSS is widely regarded as psychometrically robust, demonstrating high internal consistency. In validation studies across various populations, the scale yields Cronbach’s α coefficients typically ranging from 0.74 to 0.96, indicating excellent inter-item reliability^15^.

Because the online questionnaire required responses for all mandatory fields prior to submission, there were no missing data in the final analytical sample, precluding the need for imputation or sensitivity analyses regarding missingness.

### Statistical analysis

Data analysis was performed using SPSS, version 26. Descriptive statistics were used to summarize participant characteristics. Quantitative variables were presented as means and standard deviations (SD), while categorical variables were expressed as frequencies and percentages. Because the remission category contained relatively few participants and both remission and mild IBS represent lower symptom severity, these categories were combined for inferential analyses to provide more stable statistical comparisons. For the inferential analysis, IBS severity with its categories (remission/mild, moderate, and severe) was applied as an outcome variable. ANOVA test was used to examine whether there are statistically significant differences between means of age among three independent groups of IBS severity. The Chi-square test (χ²) was employed to examine associations between IBS severity categories and qualitative variables. To further determine the independent correlates of IBS severity, a Poisson regression model was fitted using a Generalized Linear Models (GENLIN) framework to evaluate the effect of predictors on IBS severity. The severe IBS category was coded as 1, while all other categories (remission, mild, and moderate) were combined into a single reference group and coded as 0. The independent variables entered in the Poisson regression model (anxiety, depression, obesity, drug/food hypersensitivity) were selected based on their statistical significance in the bivariate analysis (*P* < 0.05) and adjusted by age and sex as priory variables. The overall Poisson regression model was statistically significant (Likelihood Ratio χ² = 101.846, *p* < 0.001), indicating that the inclusion of the predictor variables significantly improved model fit compared with the intercept-only model. Goodness-of-fit statistics supported the adequacy of the model, with a deviance/df of 0.569 and a Pearson χ²/df of 0.838, indicating no evidence of overdispersion.

The absence of multicollinearity was assessed before fitting the regression model. A correlation matrix was constructed for all independent variables, and all Pearson correlation coefficients were less than 0.70. In addition, multicollinearity was assessed using tolerance and the variance inflation factor (VIF). The results indicated no evidence of multicollinearity, as all tolerance values were greater than 0.20 and all VIF values were below 5. The level of statistical significance was set at a P-value < 0.05.

### Ethical considerations

The current study was carried out in compliance with the ethical principles of the Declaration of Helsinki. The research protocol was reviewed and approved by the Ethics Committee of the Faculty of Medicine, Assiut University (Approval No: 04-2024-300514). Written informed consent was obtained from all participants. The confidentiality of participants’ information was strictly maintained, and no personally identifying data was included in the study.

## Results

**Fig. 1:**
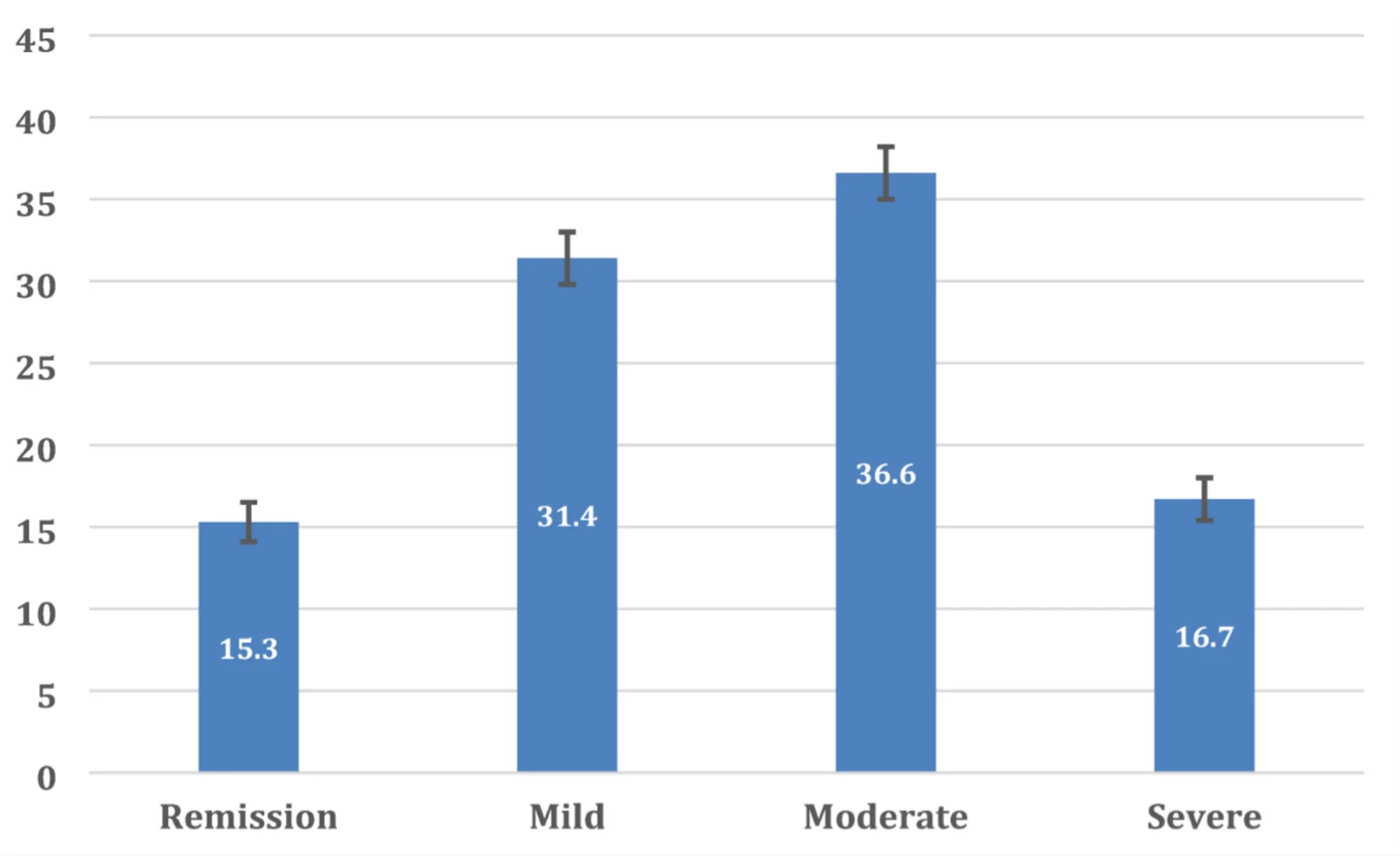
Severity of IBS among medical students in the Middle East and North Africa (*n* = 3317).

Figure 1 displays the distribution of IBS severity among the respondents. Remission, mild, moderate, and severe IBS were detected among 15.28% (95%CI 14.1–16.5), 31.38% (95%CI 29.8–33.0), 36.60% (95%CI 35.0–38.2), and 16.73% (95%CI 15.4–18.0) of the participants, respectively.

Table 1 shows the distribution of IBS severity based on personal characteristics. Students with severe IBS symptoms had a significantly higher mean age (22.20 ± 2.84) compared to those with moderate (22.00 ± 2.17) and remission/mild (21.87 ± 2.30) IBS symptoms (*p* = 0.019). Severity of IBS was not significantly associated with other sociodemographic characteristics such as gender, academic year, smoking status, marital status, parents’ marital status, or living situation (*p* > 0.05).

**Table 1:** Personal characteristics and their association with IBS severity among medical students in the Middle East and North Africa.

Variable	Total No. (%) (*n* = 3317)	Remission/mild IBS No. (%) (*n* = 1548)	Moderate IBS No. (%) (*n* = 1214)	Severe IBS No. (%) (*n* = 555)	*p*-value
Age (mean ± SD)	21.97 ± 2.36	21.87 ± 2.30	22.00 ± 2.17	22.20 ± 2.84	0.019*
Gender					
Male	893 (26.9%)	438 (49.0%)	324 (36.3%)	131 (14.7%)	0.099
Female	2424 (73.1%)	1110 (45.8%)	890 (36.7%)	424 (17.5%)	
Academic year					
First year	343 (10.3%)	173 (50.4%)	111 (32.4%)	59 (17.2%)	0.203
Second year	475 (14.3%)	210 (44.2%)	172 (36.2%)	93 (19.6%)	
Third year	652 (19.7%)	310 (47.5%)	236 (36.2%)	106 (16.3%)	
Fourth year	482 (14.5%)	216 (44.8%)	195 (40.5%)	71 (14.7%)	
Fifth year	677 (20.4%)	323 (47.7%)	256 (37.8%)	98 (14.5%)	
Sixth year	197 (5.9%)	87 (44.2%)	72 (36.5%)	38 (19.3%)	
Interns	491 (14.8%)	229 (46.6%)	168 (34.2%)	94 (19.1%)	
Smoking status					
Yes	341 (10.3%)	151 (44.3%)	135 (39.6%)	55 (16.1%)	0.478
No	2976 (89.7%)	1397 (46.9%)	1079 (36.3%)	500 (16.8%)	
History of chronic health problems					
Yes	602 (18.1%)	274 (45.5%)	230 (38.2%)	98 (16.3%)	0.664
No	2715 (81.9%)	1274 (46.9%)	984 (36.3%)	457 (16.8%)	
Marital status					
Single	3082 (92.9%)	1434 (46.5%)	1139 (37.0%)	509 (16.5%)	0.232
In relation	235 (7.1%)	114 (48.5%)	75 (31.9%)	46 (19.6%)	
Parents’ marital status					
Living together	2755 (83.1%)	1305 (47.4)	988 (35.9%)	462 (16.8%)	0.140
Not living together/divorced	241 (7.3%)	95 (39.4%)	101 (41.9%)	45 (18.7%)	
One or both parents dead	321 (9.7%)	148 (46.1%)	125 (38.9%)	48 (15.0%)	
Living situation					
With family	2572 (77.6%)	1214 (47.2%)	923 (35.9%)	435 (16.9%)	0.074
Alone	288 (8.7%)	113 (39.2%)	122 (42.4%)	53 (18.4%)	
Campus	457 (13.8%)	221 (48.4%)	169 (37.0%)	67 (14.7%)	

*: indicate significant P value.

Table 2 displays the associations of IBS severity with obesity, depression, anxiety, and different health behaviors. Among students with severe IBS symptoms, the proportion of overweight/obese (18.5%) was significantly higher than that of underweight/normal weight (16.1%) (*p* = 0.022). On the other hand, among students with remission/mild IBS symptoms, the proportion of underweight/normal weight (48.1%) was significantly higher than that of overweight/obese (42.9%). Furthermore, among students with severe IBS symptoms, there were significantly higher proportions of positive depression (*p* < 0.001), anxiety (*p* < 0.001), and history of food or drug hypersensitivity (*p* < 0.001).

**Table 2:** Association of IBS severity with obesity, depression, anxiety and different health behaviors among medical students in the Middle East and North Africa.

Variable	Total No. (%) (*n* = 3317)	Remission/mild IBS No. (%) (*n* = 1548)	Moderate IBS No. (%) (*n* = 1214)	Severe IBS No. (%) (*n* = 555)	*p*-value
Weight categories based on BMI classification					0.022*
Underweight-Normal weight	2414 (72.8%)	1161 (48.1%)	865 (35.8%)	388 (16.1%)	
Overweight/obese	903 (27.2%)	387 (42.9%)	349 (38.6%)	167 (18.5%)	
Eating junk food					0.106
Yes	2488 (75.0%)	1177 (47.3%)	914 (36.7%)	397 (16.0%)	
No	829 (25.0%)	371 (44.8%)	300 (36.2%)	158 (19.1%)	
History of food or drug hypersensitivity					< 0.001*
Yes	534 (16.1%)	207 (38.8%)	211 (39.5%)	116 (21.7%)	
No	2783 (83.9%)	1341 (48.2%)	1003 (36.0%)	439 (15.8%)	
Exercise regularly					0.148
Yes	783 (23.6%)	389 (49.7%)	268 (34.2%)	126 (16.1%)	
No	2534 (76.4%)	1159 (45.7%)	946 (37.3%)	429 (16.9%)	
Sleeping hours					0.801
< 8 h/day	1719 (51.8%)	800 (46.5%)	637 (37.1%)	282 (16.4%)	
≥ 8 h/day	1598 (48.2%)	748 (46.8%)	577 (36.1%)	273 (17.1%)	
Depression					< 0.001*
Yes	2123 (64.0%)	872 (41.1%)	797 (37.6%)	454 (21.4%)	
No	1194 (36.0%)	676 (56.6%)	409 (34.3%)	109 (9.1%)	
Anxiety					< 0.001*
Yes	2576 (77.7%)	1105 (42.9%)	972 (37.7%)	499 (19.4%)	
No	741 (22.3%)	443 (59.8%)	242 (32.7%)	56 (7.6%)	

*: indicate significant P value.

Table 3 demonstrates the Generalized linear model—Adjusted Poisson regression analysis for the correlates of severe IBS among medical students in the Middle east and North Africa. Anxious students [aPR = 1.87 (95% CI 1.418–2.461)] and depressed students [aPR = 1.86 (95% CI 1.512–2.284)] were significantly more likely to experience severe IBS symptoms (*p* < 0.001*). Similarly, students with a history of food or drug hypersensitivity were significantly more likely to have severe IBS [aPR = 1.29(95% CI 1.079–1.546)] (*p* = 0.005).

**Table 3:** Generalized linear model—Poisson regression for the correlates of severe IBS among medical students in the Middle East and North Africa.

Independent variables	Beta	S.E	aPR (95% CI)	*p*-value
Age (years)	0.031	0.017	1.031(0.998–1.066)	0.069
Gender (male)	Reference group			
Female	0.066	0.091	1.07(0.893–1.277)	0.469
History of food or drug hypersensitivity (No)	Reference group			
Yes	0.256	0.092	1.29(1.079–1.546)	0.005*
BMI (Underweight/normal weight)	Reference group			
Overweight/obese	0.098	0.083	1.103(0.937–1.298)	0.238
Anxiety (No)	Reference group			
Yes	0.625	0.141	1.87(1.418–2.461)	< 0.001*
Depression (No)	Reference group			
Yes	0.620	0.105	1.86 (1.512–2.284)	< 0.001*

*: indicate significant P value.

## Discussion

The additional analysis of the previously published cross-sectional study aimed to assess the severity of IBS among medical students in the MENA region and explore its relationship with a range of personal, behavioral, and psychological characteristics. The authors collected 3317 responses from medical students with IBS from seven different countries in the MENA region, including Egypt, Libya, Palestine, Syria, Jordan, Sudan, and Iraq^16^. The present study found that the most prevalent form of IBS was mild to moderate, while only 16.73% of the participants were affected by the severe form. This severity distribution requires careful contextualization. While general population studies utilizing the Rome IV criteria often report severe IBS manifestations in a smaller minority of patients, our finding of 16.73% severe IBS aligns with the elevated stress profile inherent to medical education. When compared to similar academic cohorts globally, this severity burden reflects a recognizable pattern; medical students in high-pressure environments—such as those reported in previous cohorts from the UAE, Bangladesh, and Iran frequently demonstrate a rightward shift toward moderate and severe symptoms compared to age-matched non-medical peers^1,11,13^. This suggests that the intense academic, clinical, and psychological demands of medical training in the MENA region not only increase the baseline prevalence of IBS but actively exacerbate its clinical severity trajectory.

Our study also revealed the age variation across the IBS severity groups; participants with severe IBS symptoms had a significantly higher mean age (22.20 ± 2.84) compared to those with moderate (22 ± 2.17) and mild (21.87 ± 2.30) symptoms. This may indicate the cumulative burden of study load stress and lifestyle modification, and its correlation with severe symptom presentation.

Algera et al. found that younger participants exhibited more severe symptoms and a lower quality of life compared to older participants^18^. This was explained by the effect of ageing on gut physiological state, as older people have a slower transit time and anorectal functional changes^18^. Beckers et al. showed that older IBS participants had a lower abdominal pain score compared to younger participants. Beckers and his colleagues reported that elderly participants had a significant reduction in visceral sensitivity, which may account for symptom attenuation^19^.

This study showed that obesity was significantly related to the severity of IBS. The proportion of obese participants with severe IBS was slightly higher than those with normal/underweight. Obesity is associated with more severe IBS symptoms, as both conditions share common underlying pathophysiological mechanisms. These overlapping factors are frequently associated with heightened gastrointestinal discomfort and a greater overall symptom burden, reflecting a complex shared pathophysiology rather than a direct unidirectional effect^20^.

Dong et al. found that there was a negative correlation between BMI, and IBS symptom severity^21^. However, Quigley et al. found that there was no significant association between BMI and symptom severity^22^. These inconsistent findings may be explained by differences in study populations, sample size, IBS diagnostic criteria, BMI classification, and adjustment for potential confounding factors. For example, variations in dietary habits, lifestyle characteristics, and the prevalence of obesity across different populations may influence the observed relationship between BMI and IBS severity. In the present study, overweight and obese students had a slightly higher proportion of severe IBS symptoms than those with normal or underweight BMI. Although the association was statistically significant in the unadjusted analysis, it was not retained in the regression model, suggesting that the relationship between BMI and IBS severity may be influenced by other factors, particularly psychological comorbidities such as anxiety and depression.

The present study revealed that participants with food/drug hypersensitivity had a higher proportion of severe IBS, and this association remained evident across both unadjusted and adjusted analyses in agreement with a previous study^23^. The observed association may reflect the heightened gastrointestinal sensitivity among individuals reporting food or drug hypersensitivity. These individuals may experience more frequent or more intense symptom exacerbations following exposure to perceived triggers, which could be associated with greater IBS symptom severity.

The literature has thoroughly explored the relationship between IBS and anxiety or depression. Several studies have found that IBS was associated with higher odds of anxiety or depression^24,25^. Others found a significant association between IBS and anxiety but not depression^22,26^.

In the present study, depression and anxiety were highly prevalent among students with severe IBS symptoms consistent with other studies^27^.

Similar results were reported by Pereyra et al., who found a significant rise in the mean IBS-SSS score in patients with depressive symptoms^28^. Moreover, IBS symptoms, such as bloating, dyspepsia, and heartburn, as well as extra-intestinal symptoms, were correlated with the severity of depression. Another study conducted in Northern India revealed that IBS severity was significantly higher among patients with psychiatric comorbidities; and depression and anxiety scores correlated positively with IBS severity^29^.

The association of anxiety or depression with severe irritable bowel syndrome symptoms can be attributed to the bidirectional gut-brain relationship, in which the symptoms of each condition amplify the other, creating a self-perpetuating cycle^30,31^. This emphasizes the substantial association between psychological distress and social environment on the progression and the severity of IBS.

Dong et al. depicted that severity of IBS symptoms was significant negative factor associated with mental health^21^. The current study demonstrated that the severity of IBS is correlated with a complex interplay of sociodemographic, behavioral, and psychiatric characteristics, each associating in distinct ways. Educational initiatives should provide students with practical guidance on evidence-based dietary modifications and healthy nutrition, as dietary habits play an important role in IBS symptom management. Psychiatric factors, particularly anxiety, and depression demonstrate a strong association with heightened symptom perception. Together, these factors create a multidimensional burden that correlates with higher IBS severity and is negatively associated with quality of life and treatment outcomes.

While these findings raise important awareness regarding the substantial burden of severe IBS and its strong correlation with psychological distress among medical students, the cross-sectional design inherently limits the study’s direct translational value for clinical practice. The present data do not provide new screening algorithms, risk stratification tools, or mechanistic insights, nor do they supply evidence that implementing policy changes would inherently improve student outcomes. Furthermore, recommending routine mental health or gastrointestinal wellness screenings may be highly impractical in resource-limited MENA settings without prior implementation science considerations or cost-effectiveness analyses. Therefore, we cautiously suggest that health services in medical schools should consider incorporating routine mental health screening, stress management programs, and accessible counseling services into student support systems to facilitate the early identification and management of students at risk of severe IBS symptoms. Moving forward, specific research priorities should include rigorous interventional trials to explicitly evaluate whether targeted screening and stress-management approaches are both feasible and clinically effective at mitigating IBS severity in medical student populations.

Furthermore, undergraduate medical education could benefit from incorporating targeted stress management and coping strategies into the curriculum. Early identification and holistic, biopsychosocial support systems are essential to support students with IBS and improve the overall well-being and academic performance of future physicians.

### Strengths and limitations

The present cross-sectional study comprised a large sample size of medical students across various countries of North Africa and the Middle East regions. In addition, IBS symptom severity was assessed by a validated screening tool. In addition, a comprehensive assessment was done to evaluate the factors associated with IBS symptom severity. This study is among the few that specifically address this objective. Despite the strength of the methodology used, this study has several limitations. Its cross-sectional design prevents causal inference regarding the observed associations between IBS severity and psychological, behavioral, or anthropometric factors. In addition, the use of self-reported online questionnaires may have introduced selection and information bias, including possible misclassification of IBS severity and related variables. Although validated instruments and Rome IV criteria were used, IBS was not confirmed by clinical evaluation, which may limit generalizability to clinically diagnosed populations. Moreover, the convenience sampling through social media may introduce selection bias, potentially excluding students with the most severe, debilitating symptoms who are less engaged online, while differentially attracting active users. Consequently, these findings may not safely generalize to all medical students within the MENA region, nor to non-medical student cohorts, rural populations, or individuals from lower socioeconomic backgrounds. Furthermore, because specific country-level identifiers were not extracted or available in the dataset for this secondary analysis, we could not apply population weighting or assess regional heterogeneity; therefore, the pooled overall estimates may be disproportionately influenced by faculties with the highest response rates. Finally, the cross-sectional data collection occurred between March and May, a period that coincides with peak academic exam seasons in several participating countries; this introduces a potential seasonal bias, capturing a localized spike in stress-induced IBS severity rather than a baseline annual average. Regarding the evaluation of hypersensitivity in the current study, there is a potential for interpretation bias, as this variable was assessed through direct self-report question rather than standardized method. Although data collection methods have been standardized, cultural, socioeconomic, and environmental factors remain significant confounders, affecting IBS severity and its associates uniquely in each country. These variations make it challenging to account for all potential confounding variables across different regions. This study should be viewed as providing observational, region-specific evidence that supports future longitudinal and interventional research, rather than advancing mechanistic or therapeutic insights.

## Conclusion

This study provides important insight into the severity of irritable bowel syndrome (IBS) among medical students in the MENA region. Although most participants reported mild to moderate IBS symptoms, a considerable proportion experienced severe symptoms. Several factors, including age, obesity, food or drug hypersensitivity, anxiety, and depression, were found to be significantly associated with IBS symptom severity, suggesting the potential association of multiple interacting factors. However, due to the cross-sectional nature of the study, causal relationships cannot be established. Further large-scale longitudinal studies are needed to better understand the complex interplay of physiological, psychological, and behavioral factors associated with IBS symptom severity.

## Supplementary Information

Below is the link to the electronic supplementary material.

Supplementary Material 1.

Supplementary Material 2.

## Data Availability

The datasets used and/or analyzed during the current study available from the corresponding author on reasonable request.
