# Supplementary material for: Severity of irritable bowel syndrome and its correlates among Middle East and North African medical students: a multicentric cross sectional study
Source: Sci Rep. 2026 Sep 9;16:28211. doi: 10.1038/s41598-026-69035-y (PMC13558663; doi:10.1038/s41598-026-69035-y)
Supplement: Supplementary file 1 [file 41598_2026_69035_MOESM1_ESM.pdf]

STROBE Statement—checklist of items that should be included in reports of observational studies

	Item No.	Recommendation	Page No.	Relevant text from manuscript
**Title and abstract**	1	(*a*) Indicate the study's design with a commonly used term in the title or the abstract	**Title** (Page 1, lines 1–2) **Abstract - Methods** (Page 1, lines 29–30)	**Title:** "...A Multicentric Cross Sectional Study" • **Abstract:** "This multicentric cross-sectional study was conducted..."
		(*b*) Provide in the abstract an informative and balanced summary of what was done and what was found	**Abstract** (Pages 1–2, lines 21–50)	
**Introduction**				
Background/rationale	2	Explain the scientific background and rationale for the investigation being reported	**Background** (Pages 3–5, lines 53–118)	IBS is framed as a biopsychosocial disorder influenced by academic stress. Medical education is highly stressful, raising IBS risk. There is a lack of large, comparable, region-specific studies on IBS severity in the MENA region
Objectives	3	State specific objectives, including any prespecified hypotheses	**Abstract / Subjects and methods** (Page 1, lines 26–28; Page 6, lines 121–125)	"...aimed to evaluate the severity of irritable bowel syndrome (IBS) and identify the correlates of severe IBS among medical students with Rome IV-confirmed IBS..."
**Methods**				
Study design	4	Present key elements of study design early in the paper	**Subjects and methods** (Page	"This study represents a secondary analysis of a previously published

			6, lines 121–125)	multicenter cross-sectional dataset..."
Setting	5	Describe the setting, locations, and relevant dates, including periods of recruitment, exposure, follow-up, and data collection	**Subjects and methods** (Page 6, lines 125–128)	"...across 27 faculties of medicine across seven countries (Egypt, Palestine, Syria, Jordan, Iraq, Libya, and Sudan) between March and May 2024"
Participants	6	(*a*) *Cohort study*—Give the eligibility criteria, and the sources and methods of selection of participants. Describe methods of follow-up<br>*Case-control study*—Give the eligibility criteria, and the sources and methods of case ascertainment and control selection. Give the rationale for the choice of cases and controls<br>*Cross-sectional study*—Give the eligibility criteria, and the sources and methods of selection of participants	**Inclusion and Exclusion Criteria** (Page 7, lines 135–139)	• **Inclusion:** Medical students identified as IBS-positive in the parent study.<br>• **Exclusion:** Prior diagnoses of IBD, GERD, GI red-flag symptoms, and family history of GI cancers
		(*b*) *Cohort study*—For matched studies, give matching criteria and number of exposed and unexposed<br>*Case-control study*—For matched studies, give matching criteria and the number of controls per case	NA	NA
Variables	7	Clearly define all outcomes, exposures, predictors, potential confounders, and effect modifiers. Give diagnostic criteria, if applicable	**Data collection tool** (Pages 6–8, lines 128, 155–170)	Self-reported online bilingual questionnaire. IBS diagnosed via **Rome IV criteria**. IBS severity assessed via the **IBS-SSS**. Anxiety assessed via **GAD-7**, and depression via **PHQ-9**.
Data sources/ measurement	8*	For each variable of interest, give sources of data and details of methods of assessment (measurement). Describe comparability of assessment methods if there is more than one group	**Data collection tool (Pages 6–8, lines 128, 155–170)**	Self-reported online bilingual questionnaire. IBS diagnosed via **Rome IV criteria**. IBS severity assessed via the **IBS-SSS**. Anxiety assessed via

				**GAD-7**, and depression via **PHQ-9**.
Bias	9	Describe any efforts to address potential sources of bias	**Data collection tool / Limitations (Pages 6–8, 18–19)**	Addressed by utilizing highly validated screening tools with robust psychometrics (IBS-SSS Cronbach's α ranges 0.74–0.96). Selection, self-report, and seasonal biases are explicitly evaluated in the limitations.
Study size	10	Explain how the study size was arrived at	**Sample Size Calculation (Page 7, lines 140–147)**	Calculated via Epi Info 7 StatCalc with 14.3% severe IBS prevalence, 99% confidence level, 5% precision, and 10% non-response, requiring a minimum of 358 participants. All eligible IBS-positive students (n = 3,317) were included.

Continued on next page

Quantitative variables	11	Explain how quantitative variables were handled in the analyses. If applicable, describe which groupings were chosen and why	**Statistical analysis** (Pages 8–9, lines 174–180)	Age was handled as mean ± SD. BMI was categorized. Remission (<75) and mild (75 to <175) IBS-SSS score groups were combined into a "remission/mild" category for inferential analysis due to low sample size in remission
Statistical methods	12	(*a*) Describe all statistical methods, including those used to control for confounding	**Statistical analysis** (Pages 8–9, lines 174–199)	Descriptive stats (frequencies, means, SD). ANOVA was used for mean age; Chi-square for associations with qualitative variables. A **Generalized Linear Model — Poisson regression framework** was fitted to evaluate predictors of severe IBS, adjusting for age and sex as a priori confounders
		(*b*) Describe any methods used to examine subgroups and interactions	**Study site, design and population** (Page 6, lines 130–133)	Analysis was restricted specifically to the subgroup of medical students classified as IBS-positive (n = 3,317) from the larger parent dataset.
		(*c*) Explain how missing data were addressed	**Data collection tool** (Page 8, lines 171–173)	Zero missing data: "Because the online questionnaire required responses for all mandatory fields prior to submission, there were no missing data...".
		(*d*) *Cohort study*—If applicable, explain how loss to follow-up was addressed<br>*Case-control study*—If applicable, explain how matching of cases and controls was addressed	**Sampling technique** (Page 7,	Multistage approach: random sampling of 27 universities, followed by convenience sampling

		Cross-sectional study—If applicable, describe analytical methods taking account of sampling strategy	lines 148–154)	via social media. Complete coverage was used for the IBS-positive cohort.
		(*e*) Describe any sensitivity analyses	**Data collection tool** (Page 8, lines 171–173)	Precluded/unnecessary because there was zero missing data in the final sample.
**Results**				
Participants	13*	(a) Report numbers of individuals at each stage of study—eg numbers potentially eligible, examined for eligibility, confirmed eligible, included in the study, completing follow-up, and analysed	**Subjects and methods / Results** (Pages 6–7, 10)	Parent study recruited 8,275 medical students. Analysis was restricted to the 3,317 participants confirmed as IBS-positive. Figure 1 displays the final analyzed severity distribution.
		(b) Give reasons for non-participation at each stage	NA	NA
		(c) Consider use of a flow diagram	NA	NA
Descriptive data	14*	(a) Give characteristics of study participants (eg demographic, clinical, social) and information on exposures and potential confounders	**Results** (Pages 11–13, Tables 1 and 2)	**Table 1** outlines age, gender, academic year, smoking, marital status, and living situation. **Table 2** details BMI weight categories, eating behaviors, exercise, sleeping hours, anxiety, and depression.
		(b) Indicate number of participants with missing data for each variable of interest	**Data collection tool** (Page 8, lines 171–173)	No missing data for any of the analyzed variables.
		(c) *Cohort study*—Summarise follow-up time (eg, average and total amount)	NA	NA
Outcome data	15*	*Cohort study*—Report numbers of outcome events or summary measures over time	*NA*	*NA*
		Case-control study—Report numbers in each exposure category, or summary measures of exposure	*NA*	*NA*

		Cross-sectional study—Report numbers of outcome events or summary measures	**Results / Figure 1** (Page 10, lines 207–209)	**Summary measures of IBS severity:** Remission = 15.28%, Mild = 31.38%, Moderate = 36.60%, Severe = 16.73%.
Main results	16	(*a*) Give unadjusted estimates and, if applicable, confounder-adjusted estimates and their precision (eg, 95% confidence interval). Make clear which confounders were adjusted for and why they were included	**Results** (Page 13, Table 3)	Reports adjusted prevalence ratios (aPR) with 95% Confidence Intervals. Independent correlates of severe IBS: **Anxiety** [aPR = 1.87 (95% CI: 1.418–2.461)], **Depression** [aPR = 1.86 (95% CI: 1.512–2.284)], and **Hypersensitivity** [aPR = 1.29 (95% CI: 1.079–1.546)].
		(*b*) Report category boundaries when continuous variables were categorized	**Data collection tool** (Page 8, lines 166–168)	IBS-SSS total scores categorized into: remission (<75), mild (75 to <175), moderate (175 to <300), and severe (≥300) .
		(*c*) If relevant, consider translating estimates of relative risk into absolute risk for a meaningful time period	NA	NA

Continued on next page

Other analyses	17	Report other analyses done—eg analyses of subgroups and interactions, and sensitivity analyses	Results (Pages 11–13, Tables 1 and 2)	Bivariate associations (using Chi-square and ANOVA) between personal/behavioral characteristics and IBS severity.
**Discussion**				
Key results	18	Summarise key results with reference to study objectives	**Discussion** (Pages 14–15, lines 244–263)	Summarizes that mild-to-moderate IBS was most common, while 16.73% had severe symptoms. The higher severity burden is directly linked to the cumulative stress of medical training.
Limitations	19	Discuss limitations of the study, taking into account sources of potential bias or imprecision. Discuss both direction and magnitude of any potential bias	Strengths and Limitations (Pages 18–19, lines 337–359)	Discusses cross-sectional design limits (no causal inference), selection and self-report biases, lack of clinical diagnostic confirmation, convenience sampling bias, lack of regional weighting, seasonal bias (exam season), and potential interpretation bias of self-reported hypersensitivity.
Interpretation	20	Give a cautious overall interpretation of results considering objectives, limitations, multiplicity of analyses, results from similar studies, and other relevant evidence	Discussion / Conclusion (Pages 2, 17–19, lines 314–332, 362–370)	Concludes that more than half of students suffer moderate-to-severe symptoms, strongly tied to psychological distress . Cautiously suggests implementing counseling, stress management, and mental health screenings in medical schools.
Generalisability	21	Discuss the generalisability (external validity) of the study results	Strengths and Limitations	Generalizability may be limited; findings may not generalize to non-medical students, rural populations,

			(Page 19, lines 345–347)	or individuals from lower socioeconomic backgrounds in the MENA region.
**Other information**				
Funding	22	Give the source of funding and the role of the funders for the present study and, if applicable, for the original study on which the present article is based	Funding (Page 19, lines 371–372)	"No funding was obtained in the present study"

*Give information separately for cases and controls in case-control studies and, if applicable, for exposed and unexposed groups in cohort and cross-sectional studies.

Note: An Explanation and Elaboration article discusses each checklist item and gives methodological background and published examples of transparent reporting. The STROBE checklist is best used in conjunction with this article (freely available on the Web sites of PLoS Medicine at http://www.plosmedicine.org/, Annals of Internal Medicine at http://www.annals.org/, and Epidemiology at http://www.epidem.com/). Information on the STROBE Initiative is available at www.strobe-statement.org.
